# Data mining reveal the association between diabetic foot ulcer and peripheral artery disease

**DOI:** 10.3389/fpubh.2022.963426

**Published:** 2022-08-18

**Authors:** Jie Zou, Wen Zhang, Xiaoming Chen, Wenxing Su, Daojiang Yu

**Affiliations:** ^1^Department of Cosmetic Plastic and Burn Surgery, The First Affiliated Hospital of Chengdu Medical College, Chengdu, China; ^2^Department of Plastic and Burn Surgery, The Second Affiliated Hospital of Chengdu Medical College, China National Nuclear Corporation 416 Hospital, Chengdu, China; ^3^School of Clinical Medicine, Chengdu Medical College, Chengdu, China; ^4^Department of Dermatology, The First Affiliated Hospital of Chengdu Medical College, Chengdu, China

**Keywords:** diabetic foot ulcer, peripheral artery disease, bioinformatics, differentially expressed genes, hub genes

## Abstract

**Background:**

Diabetic foot ulcer (DFU) and peripheral artery disease (PAD) are common diseases that seriously affect the quality of life and bring a huge economic burden to society. Although mounting evidence supports a close link between the two disorders, the mechanisms of comorbidity remain to be fully elucidated.

**Methods:**

The gene expression profiles of DFU (GSE80178) and PAD (GSE100927) were downloaded from the Gene Expression Omnibus (GEO) database. Gene Ontology (GO) and Kyoto Encyclopedia of Genes and Genomes (KEGG) performed pathway enrichment analysis for common differentially expressed genes (DEGs) present in DFU and PAD. Subsequently, we constructed a protein-protein interaction (PPI) network using the STRING database and detected core modules and hub genes in the network. Finally, we analyzed the co-expression network and the TF-miRNA-mRNA regulatory network of hub genes.

**Results:**

A total of 167 common DEGs (91 up-regulated genes and 76 down-regulated genes) was selected for subsequent analyses. Functional analysis emphasizes the important role of chemokines and cytokines in these two diseases. Finally, six hub genes were identified using cytoHubba, including CXCL8, IL1RN, MMP1, CD68, CCR7 and CCL3.

**Conclusions:**

The hub genes and signaling pathways involved can regulate both diseases simultaneously, suggesting a close relationship between the molecular mechanisms of the two diseases and possible targets for drugs that intervene in both diseases.

## Introduction

Foot infections and ulcers of patients with diabetes (DFU) are the major complications of diabetes and tightly associated with the morbidity and serious adverse events (1). The prevalence and incidence of DFU is 4–10 and 2.4–2.6%, respectively (2). Almost half of patients suffer from PAD, which increases the risk of infection, non-healing ulcers and amputations (3). Atherosclerosis is a chronic inflammatory disease which caused by the increased low-density lipoprotein cholesterol in the circulation. The development of atherosclerosis leads to the strokes, ischemic heart disease and PAD (4).

DFU and PAD possess some common risk factors such as hypertension, obesity, cigarette smoking, deficiency of insulin secretion or insulin resistance (2). PAD promotes the progression of DFU by increasing the risk of infection and non-healing ulcers (5, 6). Patients with DFU and PAD have the higher mortality rates, major amputation rates and slower healing (7). Cai et al. reported that serum IL1β level was significantly increased in patients with diabetic lower extremity arterial diseases compared to the diabetes patients without lower extremity arterial diseases (8). Due to the close association of DFU and PAD and some common metabolic and immune-related factors, some molecular mechanisms might be involved in the development and progression of DFU and PAD. Previously studies have shown that the innate immune and adaptive immune response, such as the abnormal secretion of cytokines and the alteration of lymphocytes subpopulations, promote the progression of both diseases (2).

**Figure 1:**
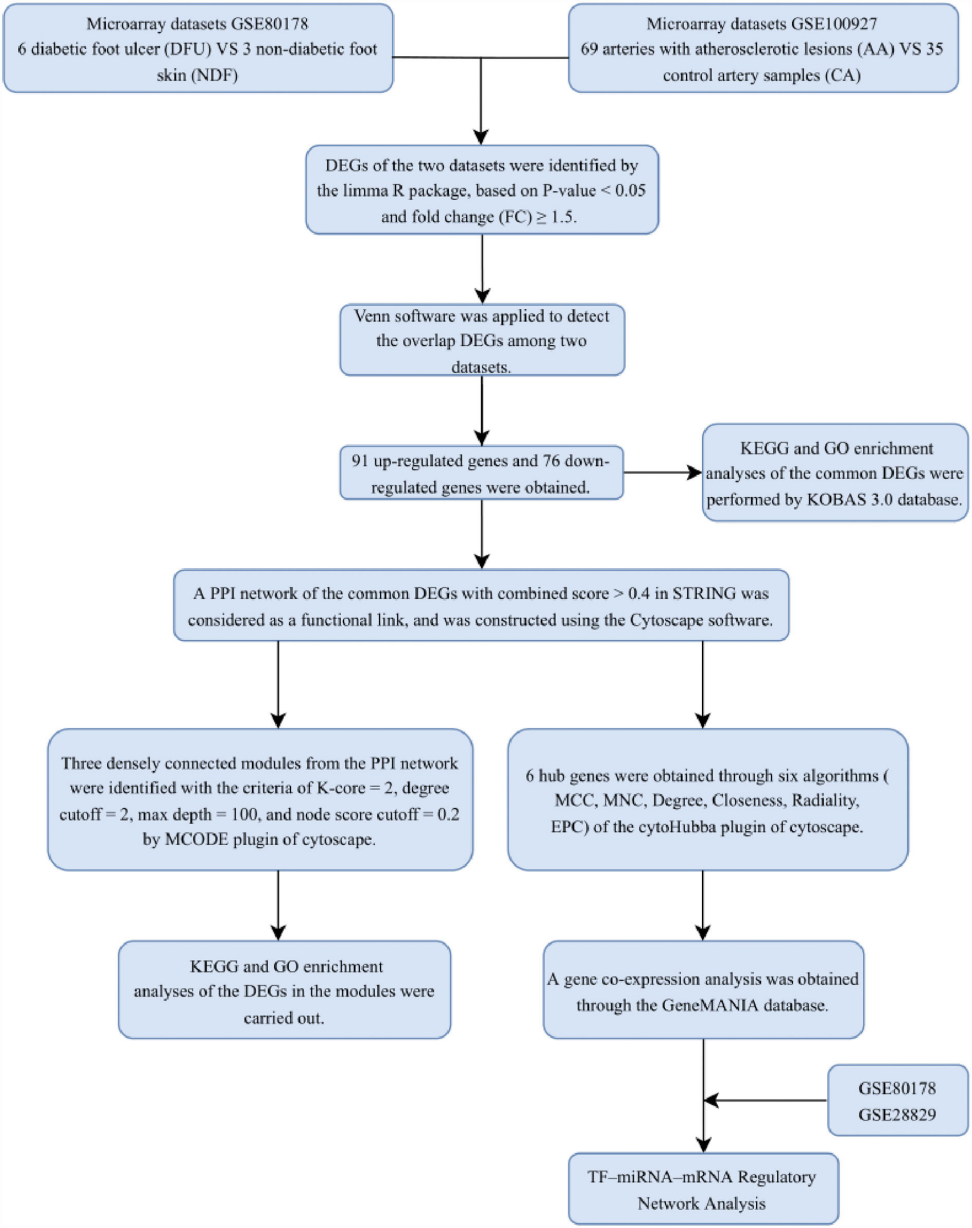
Research design flow chart.

With the development of transcriptomics and bioinformatics, the common transcription feature may provide some new ideas for the pathogenesis of DFU and PAD. This study was designed to identify the common genes between patients with DFU and PAD. The two gene expression data sets to (GSE80178) and (GSE100927) were downloaded from the GEO database. The common DEGs and their functions in DFU and PAD were analyzed. In addition, we identified the gene modules and the hub genes by constructing the PPI network. Finally, six hub genes were identified, and transcription factor (TF) and miRNA were also traced based on the hub genes in the PPI network. The research flowchart of this research was shown in Figure 1.

## Materials and methods

### Raw data collection

GEO (http://www.ncbi.nlm.nih.gov/geo) (9) is a public database containing a large number of high-throughput sequencing and microarray data sets submitted by research institutes worldwide. We searched for related gene expression datasets using diabetic foot and atherosclerosis as keywords. The inclusion criteria are set as: two independent expression profiles contain the largest sample size and the test specimens included should be from humans. Finally, two microarray datasets [SE80178 (10) and GSE100927 (11)] were downloaded based on the Affymetrix GPL16686 platform and Affymetrix GPL17077 platform. The GSE80178 dataset contains 6 diabetic foot ulcer (DFU) and 3 non-diabetic foot skin (NDF). GSE100927 consists of 69 peripheral arteries with atherosclerotic lesions (AA) and 35 control artery samples (CA).

### Identification of DEGs

Limma package (version: 3.40.2) of R software was used to study the differential expression of mRNAs. The *P*-value was analyzed to correct for false positive results in GEO datasets. “*P* < 0.05 and Fold Change (FC) ≥ 1.5” were defined as the thresholds for the screening of differential expression of mRNAs. Probe sets with no corresponding gene symbols or genes with more than one probe set were removed or averaged, respectively. The online Venn diagram tool (https://bioinfogp.cnb.csic.es/tools/venny/index.html) was used to obtain their overlap DEGs.

### Enrichment analyses of DEGs

KEGG Orthology Based Annotation System (KOBAS) (http://kobas.cbi.pku.edu.cn) (12) is a Web server for gene/protein functional annotation and functional enrichment developed by Peking University, which collects 4,325 species functional annotation information. In order to better understand the main biological functions of DEGs, we used the KOBAS 3.0 to analyze the GO and KEGG pathways that up-regulate and down-regulate DEGs. Adjusted *P*-value < 0.05 was considered significant.

### PPI network construction and module analysis

Search Tool for the Retrieval of Interacting Genes (STRING; http://string-db.org) (version 11.5) (13) can search for the relationship between proteins of interest, such as direct binding relationships, or coexisting upstream and downstream regulatory pathways, to construct a PPI network with complex regulatory relationships. Interactions with a combined score over 0.4 were considered statistically significant. Cytoscape (http://www.cytoscape.org) (version 3.9.0) (14) was used to visualize this PPI network. Cytoscape's plug-in molecular complex detection technology (MCODE) was used to analyze key functional modules. Set the selection criteria as: K-core = 2, degree cutoff = 2, max depth = 100, and node score cutoff = 0.2. Then the KEGG and GO analysis of the involved modular genes were performed with KOBAS 3.0.

### Selection and analysis of hub genes

The hub genes were identified by using the cytoHubba plug-in of Cytoscape. Here, we used six common algorithms (MCC, MNC, Degree, Closeness, Radiality, EPC) to evaluate and select hub genes. Subsequently, we constructed a co-expression network of these hub genes *via* GeneMANIA (http://www.genemania.org/) (15), which is a reliable tool for identifying internal associations in gene sets. Given a query list, GeneMANIA extends the list with functionally similar genes that it identifies using available genomics and proteomics data.

### TF-MiRNA-MRNA regulatory network analysis

To further understand the regulatory mechanism of hub genes, TF-target interactions were obtained through the Transcriptional Regulatory Relationships Unraveled by Sentence-based Text mining (TRRUST) (16). TRRUST is a database for the prediction of transcriptional regulatory networks, which contains the target genes corresponding to TFs and the regulatory relationships between TFs. In addition, miRNA-target interactions were obtained by Mirwalk, which is a publicly available database that focuses on miRNA-target interactions (17). In order to improve the accuracy, the screening condition was set as: the predicted miRNA has been verified by experiments. Finally, miRNA-target interactions and TF-target interactions were integrated to construct the TF-miRNA-mRNA regulatory network by Cytoscape.

### Validation of hub genes

To confirm the reliability of our results, hub genes expression was verified by Student's t test. Since GSE80178 is the only dataset that contains DFU in the GEO database, we choose it for validation. Additionally, GSE28829 consists of 13 early atherosclerotic plaque samples (EA) and 16 AA is also used for verification. *P*-value < 0.05 was considered statistically significant.

## Results

### Identification of DEGs

**Figure 2:**
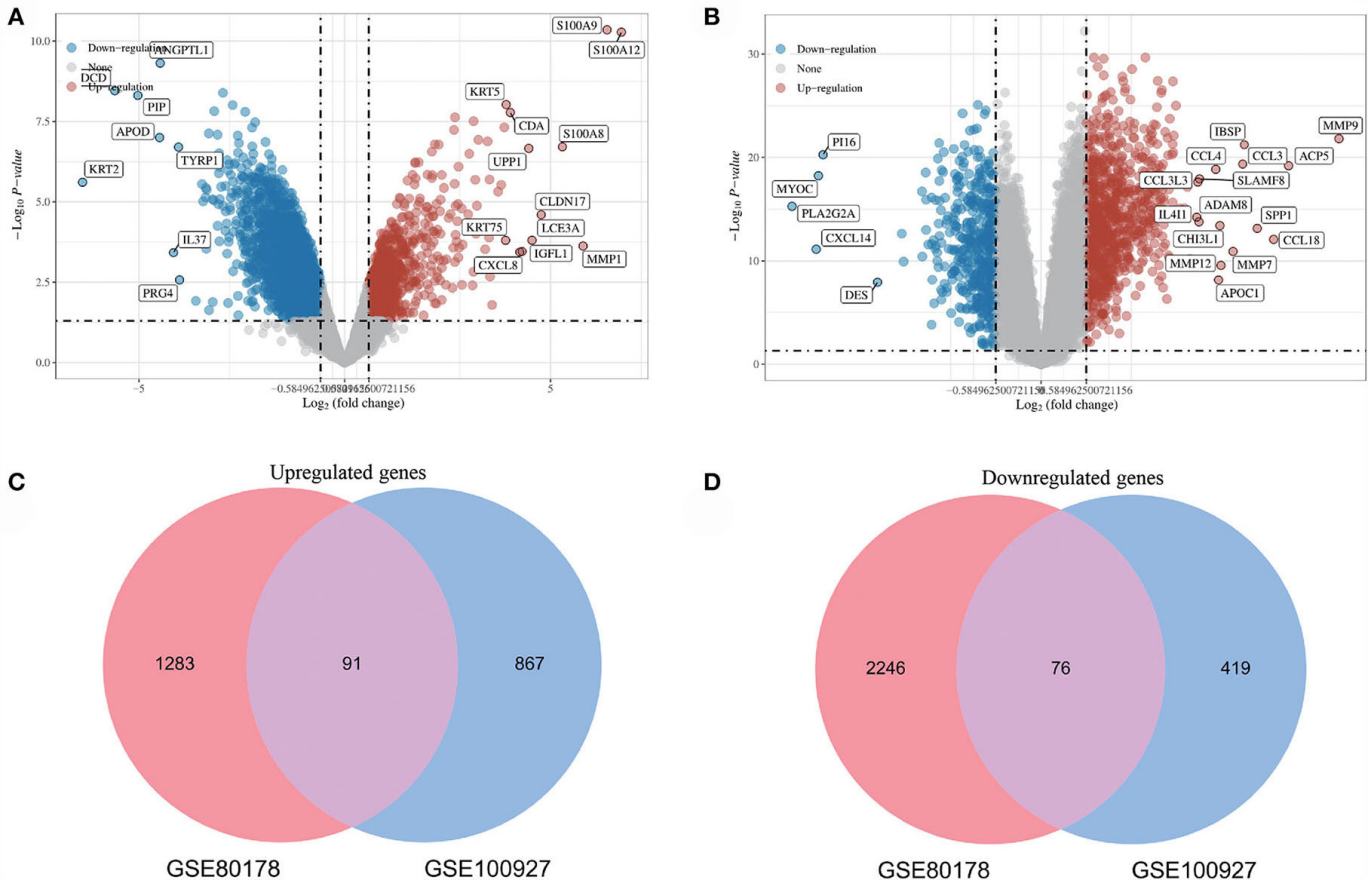
Volcano diagram and Venn diagram. **(A)** The volcano map of GSE80178. **(B)** The volcano map of GSE100927. Upregulated genes are marked in light red; downregulated genes are marked in light blue. **(C,D)** The two datasets showed an overlap of 167 DEGs, including 91 up-regulated genes and 76 down-regulated genes.

After standardizing the microarray results, DEGs (3696 in GSE80178 and 1453 in GSE100927) were identified (Figures 2A,B). Through Venn diagram calculation, we obtained 91 overlapping up-regulated genes and 76 overlapping down-regulated genes in GSE80178 and GSE100927 (Figures 2C,D). Supplementary Table S1 shows the detailed information of these overlapping DEGs.

### Analysis of the functional characteristics of common DEGs

**Figure 3:**
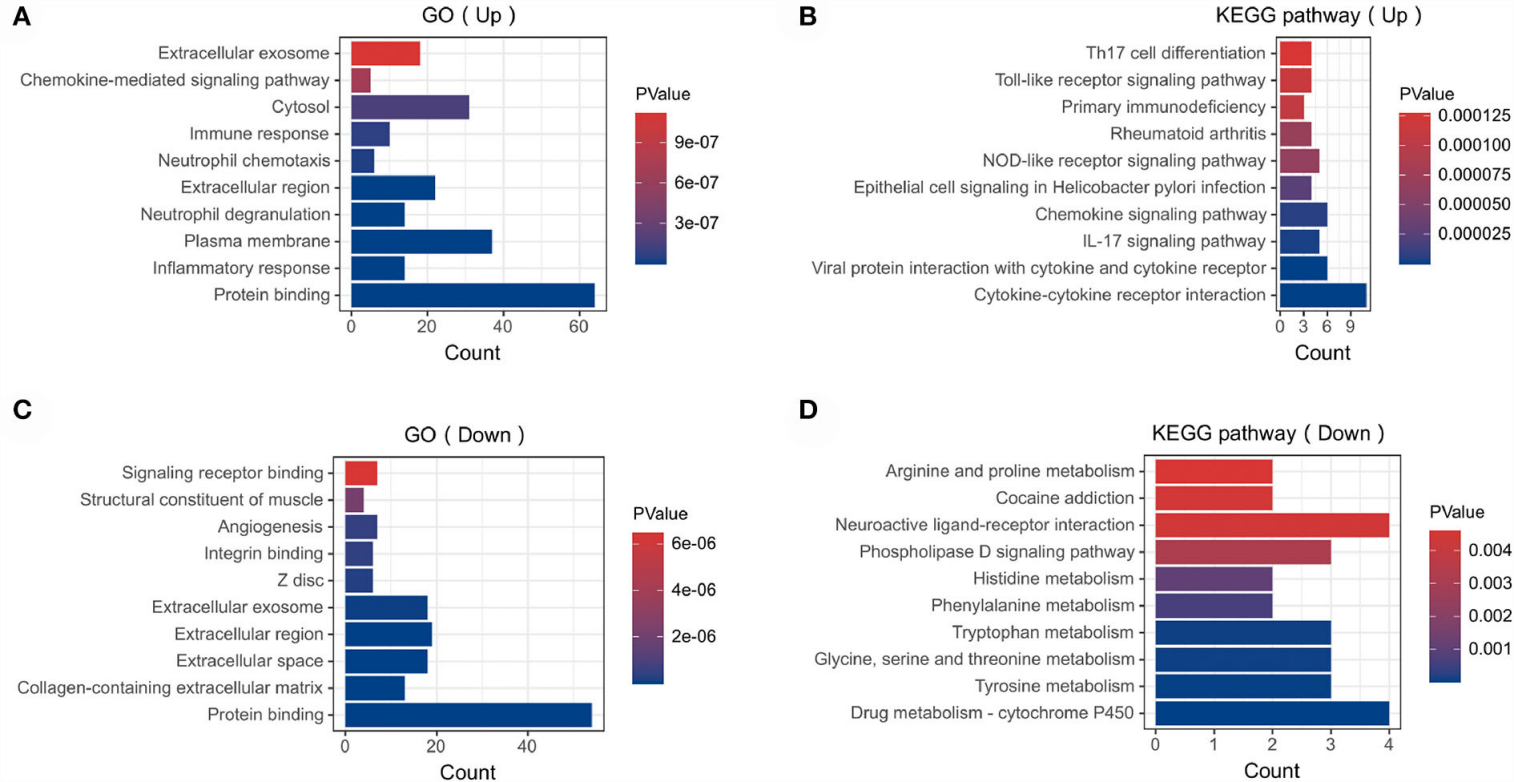
Functional enrichment: **(A)** enrichment result of up-regulated DEGs GO term. **(B)** enrichment result of up-regulated DEGs KEGG pathway **(C)** enrichment result of down-regulated DEGs GO term **(D)** enrichment result of down-regulated DEGs KEGG pathway. The result of functional enrichment comes from the KOBAS database.

In order to analyze the biological functions and pathways involved in these overlapping genes, GO and KEGG Pathway enrichment analysis were performed. GO analysis results show that these up-regulated genes were mainly enriched in protein binding (*P* =1.19E-12), inflammatory response (*P* = 1.81E-10), plasma membrane (P = 5.82E-10) and neutrophil degranulation (P = 1.91E-09) (Figure 3A). These down-regulated genes were mainly enriched in protein binding (P = 1.96E-10), collagen-containing extracellular matrix (P = 2.31E-10), extracellular space (P = 3.09E-07) and angiogenesis (*P* = 5.07E-07) (Figure 3C). In terms of KEGG Pathway, these up-regulated genes were mainly enriched in cytokine-cytokine receptor interaction (P = 1.65E-08), viral protein interaction with cytokine and cytokine receptor (*P* = 1.15E-05), IL17 signaling pathway (P = 1.44E-04) and chemokine signaling pathway (P = 2.09E-04) (Figure 3B). These down-regulated genes were mainly enriched in drug metabolism–cytochrome P450 (*P* = 1.29E-3), tyrosine metabolism (*P* = 2.02E-3), glycine, serine and threonine metabolism (*P* = 2.02E-3) and tryptophan metabolism (*P* = 2.02E-3) (Figure 3D).

### PPI network construction and module analysis

**Figure 4:**
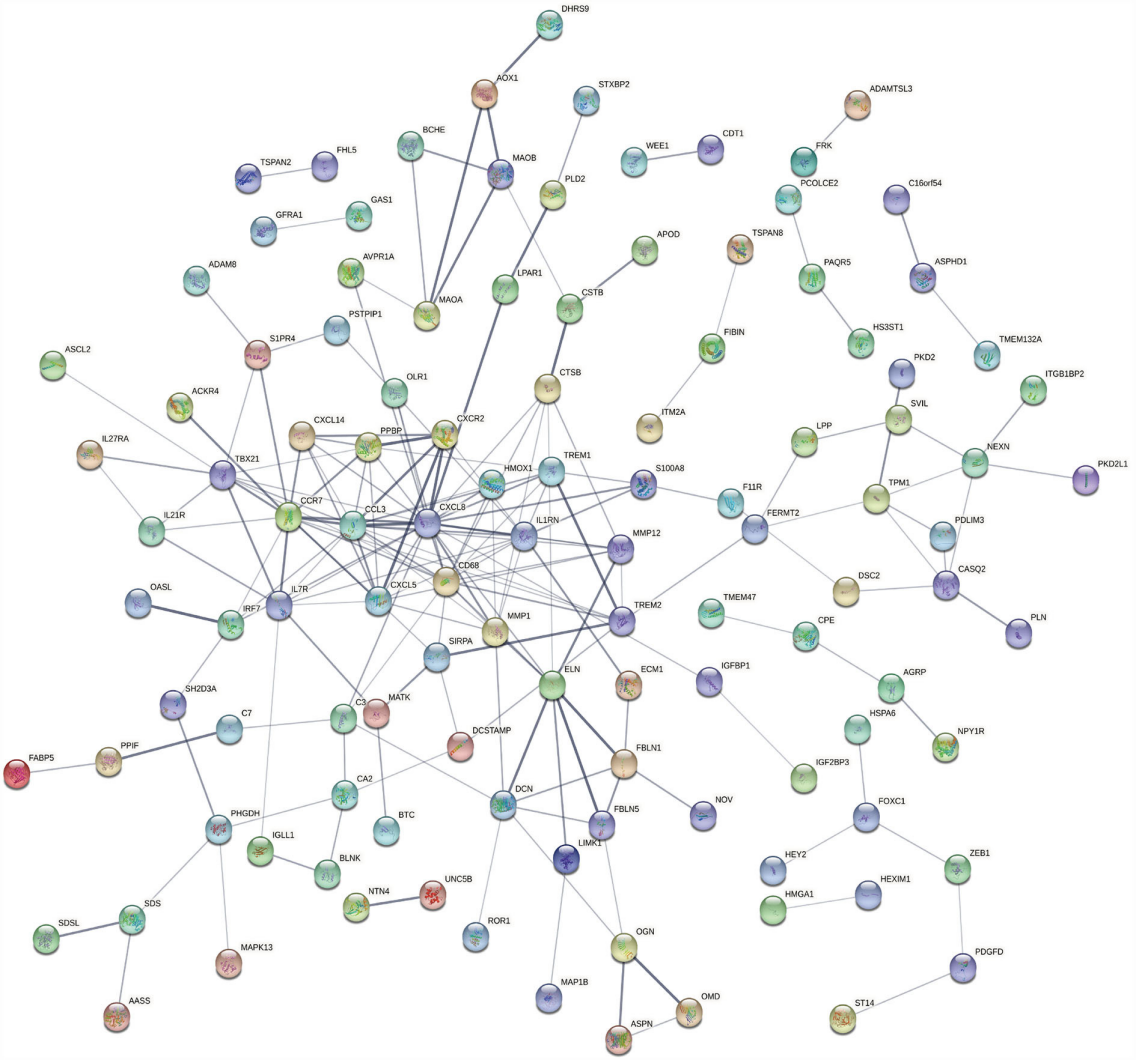
PPI network constructed using the STRING database.

**Figure 5:**
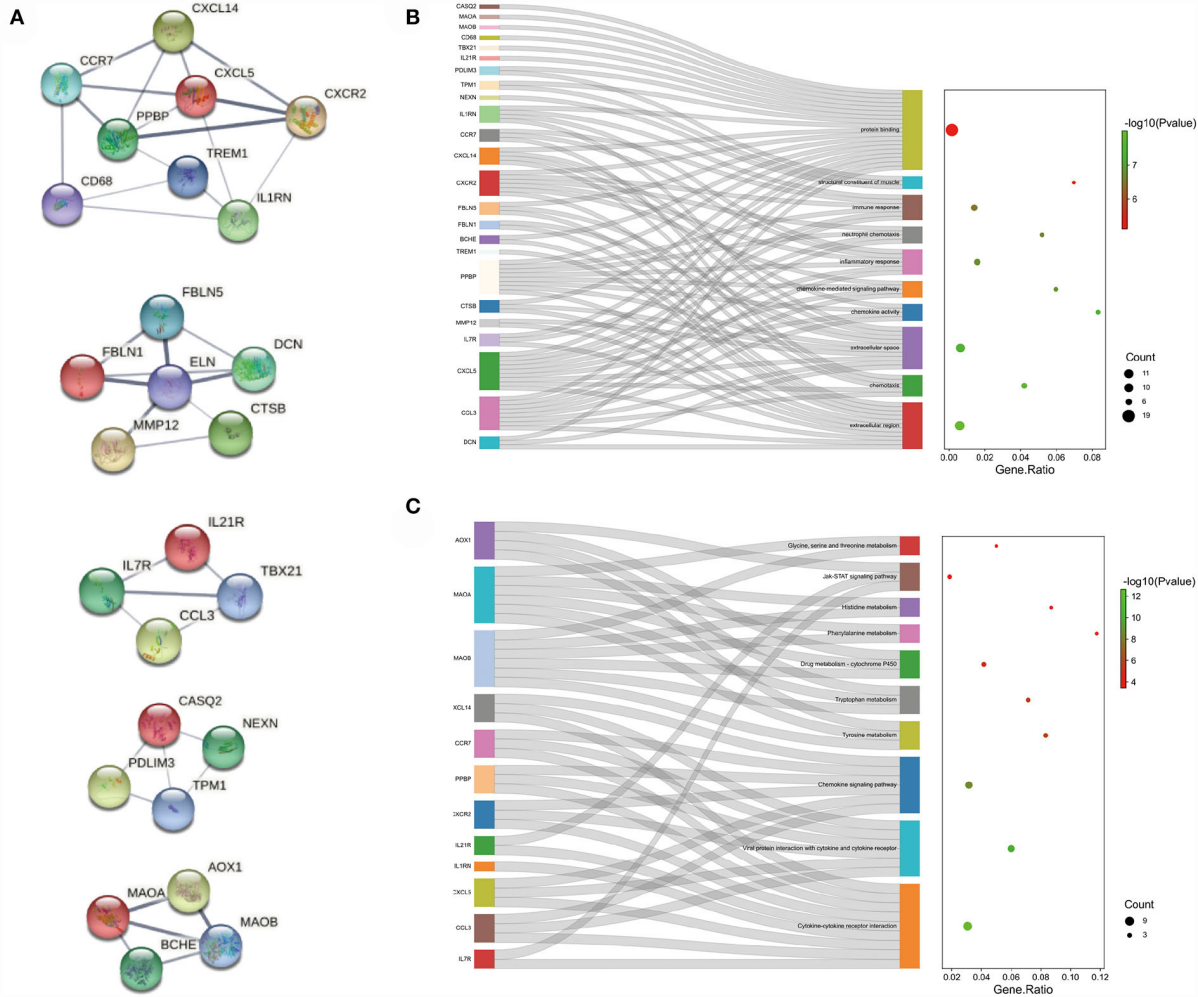
Significant gene module and enrichment analysis of the modular genes **(A)** Five significant gene clustering modules. **(B,C)** GO and KEGG enrichment analysis of the modular genes. The size of the circle represents the number of genes involved, and the abscissa represents the frequency of the genes involved in the term total genes.

The PPI network of the overlapping DEGs with combined scores > 0.4 was constructed using Cytoscape, which contained 114 nodes and 190 interaction pairs (Figure 4). Three closely connected gene modules were obtained through MCODE plug-in of Cytoscape, including 26 common DEGs and 40 interaction pairs (Figure 5A). GO analysis showed that these genes are related to extracellular region, chemotaxis, extracellular space and chemokine activity (Figure 5B). KEGG Pathway analysis showed that them to be mainly involved in cytokine-cytokine receptor interaction, viral protein interaction with cytokine and cytokine receptor, and chemokine signaling pathway (Figure 5C).

### Selection and analysis of hub genes

**Figure 6:**
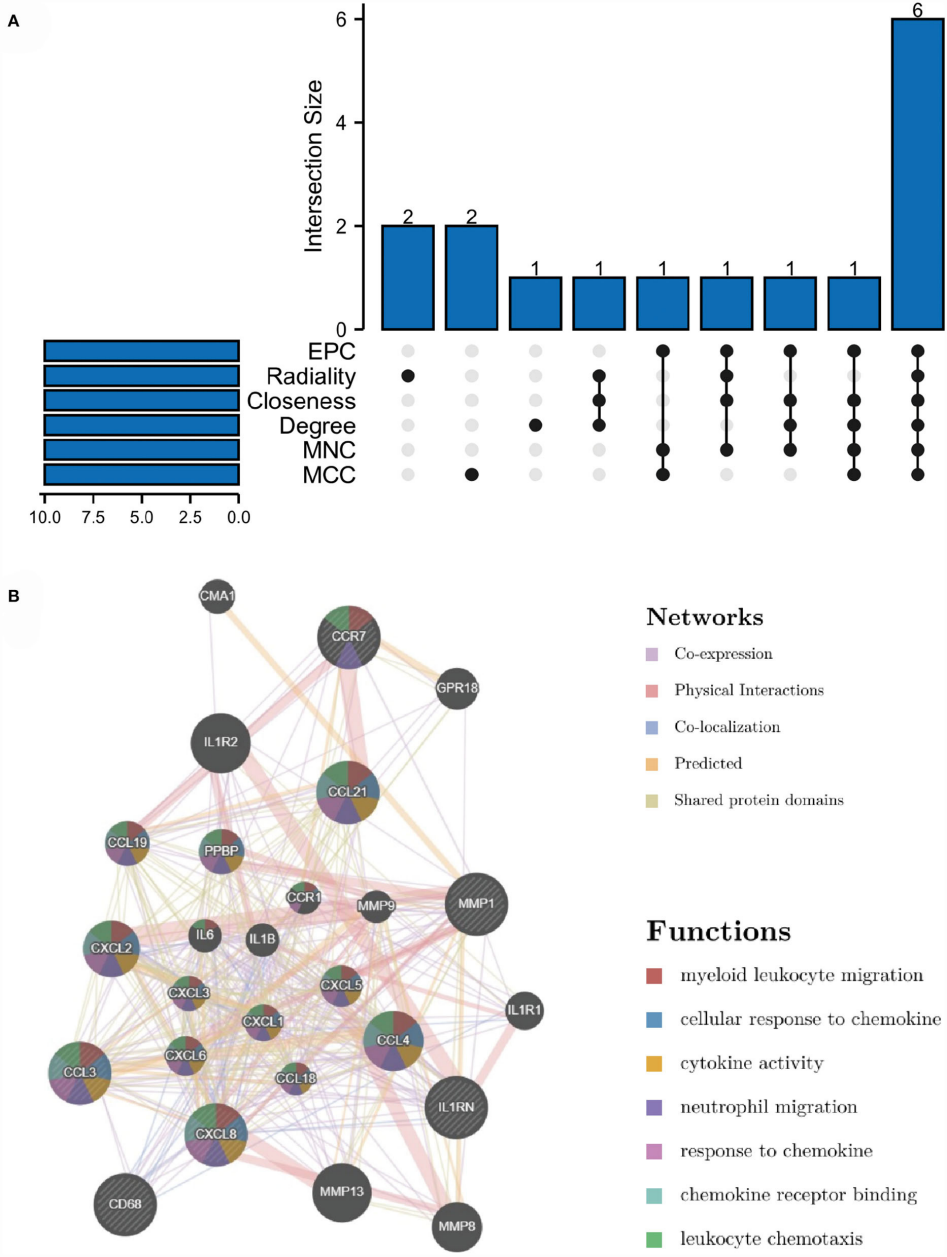
Venn diagram and co-expression network of hub genes. **(A)** The Venn diagram showed that six algorithms have screened out 6 overlapping hub genes. **(B)** Hub genes and their co-expression genes were analyzed *via* GeneMANIA.

Through the six algorithms of plug-in cytoHubba, we have calculated the top 10 hub genes (Table 1). After taking the intersection of the Venn diagrams, we found six overlapping hub genes, including CXCL8, IL1RN, MMP1, CD68, CCR7 and CCL3 (Figure 6A). Table 2 shows their full names and related functions. Based on the GeneMANIA database, we analyzed the co-expression network and related functions of these genes. These genes showed the complex PPI network with the co-expression of 66.19%, physical interactions of 11.4%, co-localization of 10.44%, predicted of 7.65% and shared protein domains of 4.26% (Figure 6B). These genes are related to cellular response to chemokine, cytokine activity and response to chemokine (Figure 6B).

**Table 1:** The top 10 hub genes rank in cytoHubba.

**MCC**	**MNC**	**Degree**	**Closeness**	**Radiality**	**EPC**
IL1RN	IL1RN	IL1RN	IL1RN	C3	IL1RN
CXCL8	CXCL8	CXCL8	CXCL8	TREM2	CXCL8
CD68	CD68	CD68	CD68	IL1RN	CD68
CXCL5	CXCL5	CXCL5	CXCL5	CD68	CXCL5
PPBP	MMP12	TBX21	MMP12	CXCL8	MMP12
CCL3	TBX21	IL7R	TBX21	MMP12	TBX21
CXCR2	PPBP	CCL3	CCL3	CCL3	PPBP
MMP1	CCL3	MMP1	MMP1	CCR7	CCL3
CCR7	MMP1	CCR7	CCR7	MMP1	MMP1
CXCL14	CCR7	ELN	ELN	ELN	CCR7

**Table 2:** The details of the hub genes.

**No**.	**Gene symbol**	**Full name**	**Function**
1	CXCL8	C-X-C Motif Chemokine Ligand 8	The protein encoded by this gene is a member of the CXC chemokine family and is a major mediator of the inflammatory response. The encoded protein is secreted primarily by neutrophils, where it serves as a chemotactic factor by guiding the neutrophils to the site of infection. This chemokine is also a potent angiogenic factor.
2	IL1RN	Interleukin 1 Receptor Antagonist	The protein encoded by this gene is a member of the interleukin 1 cytokine family. This protein inhibits the activities of interleukin 1, alpha (IL1A) and interleukin 1, beta (IL1B), and modulates a variety of interleukin 1 related immune and inflammatory responses.
3	MMP1	Matrix Metallopeptidase 1	This gene encodes a member of the peptidase M10 family of matrix metalloproteinases (MMPs). Proteins in this family are involved in the breakdown of extracellular matrix in normal physiological processes, such as embryonic development, reproduction, and tissue remodeling, as well as in disease processes, such as arthritis and metastasis.
4	CD68	CD68 Molecule	It is a member of the lysosomal/endosomal-associated membrane glycoprotein (LAMP) family. The protein primarily localizes to lysosomes and endosomes with a smaller fraction circulating to the cell surface. It is a type I integral membrane protein with a heavily glycosylated extracellular domain and binds to tissue- and organ-specific lectins or selectins.
5	CCR7	C-C Motif Chemokine Receptor 7	The chemokine (C-C motif) ligand 19 (CCL19/ECL) has been reported to be a specific ligand of this receptor. Signals mediated by this receptor regulate T cell homeostasis in lymph nodes, and may also function in the activation and polarization of T cells, and in chronic inflammation pathogenesis.
6	CCL3	C-C Motif Chemokine Ligand 3	The encoded protein, also known as macrophage inflammatory protein 1 alpha, plays a role in inflammatory responses through binding to the receptors CCR1, CCR4 and CCR5.

### TF-MiRNA-MRNA regulatory network analysis

**Figure 7:**
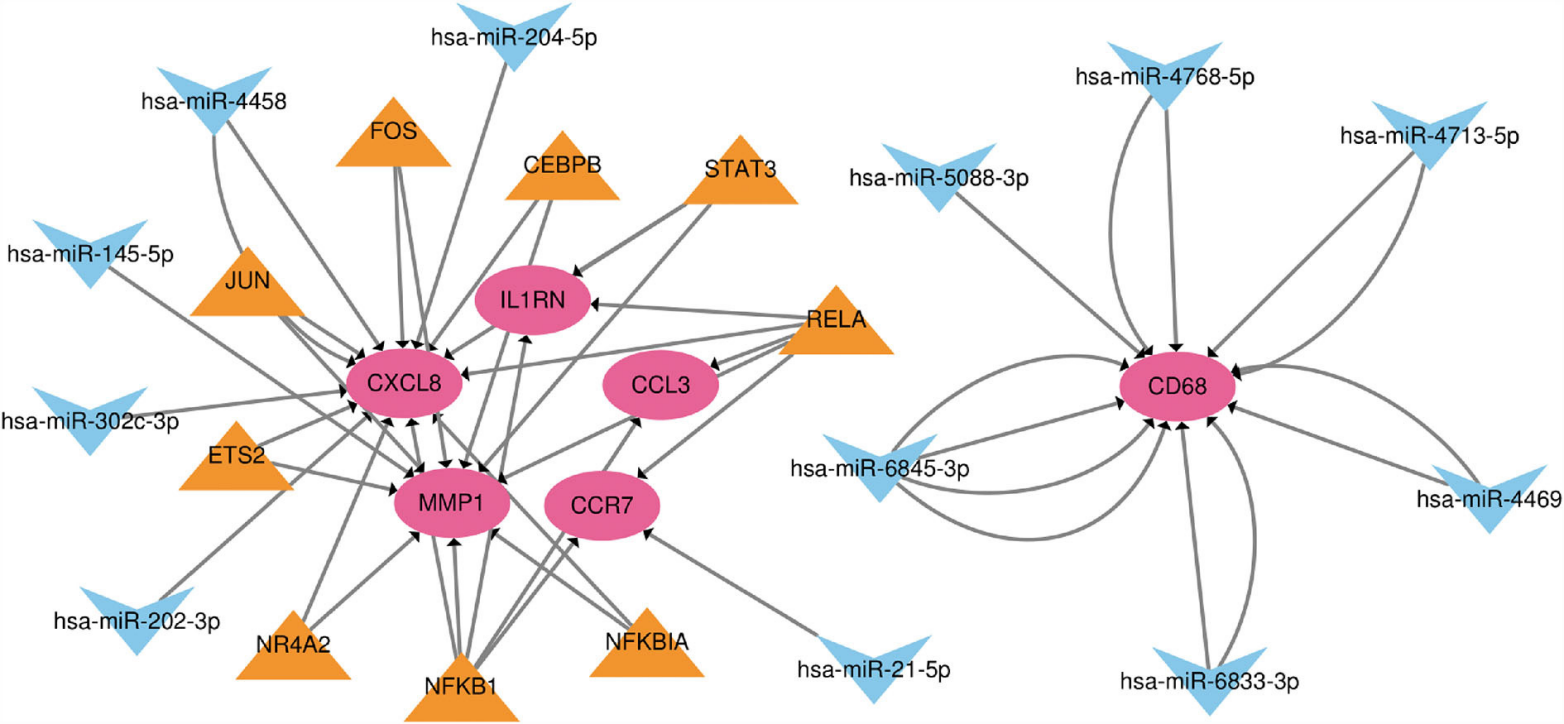
The TF-miRNA-mRNA regulatory network. Red nodes represent hub genes, blue inverted triangles represent miRNAs and yellow triangles represent TFs.

Based on the TRRUST and Mirwalk database, we found that 7 TFs and 12 miRNA may regulate the expression of these genes. 21 miRNA-mRNA pairs and 25 TF-mRNA pairs were integrated to structure a TF-miRNA-mRNA regulatory network (Figure 7).

### Validation of hub genes expression

**Figure 8:**
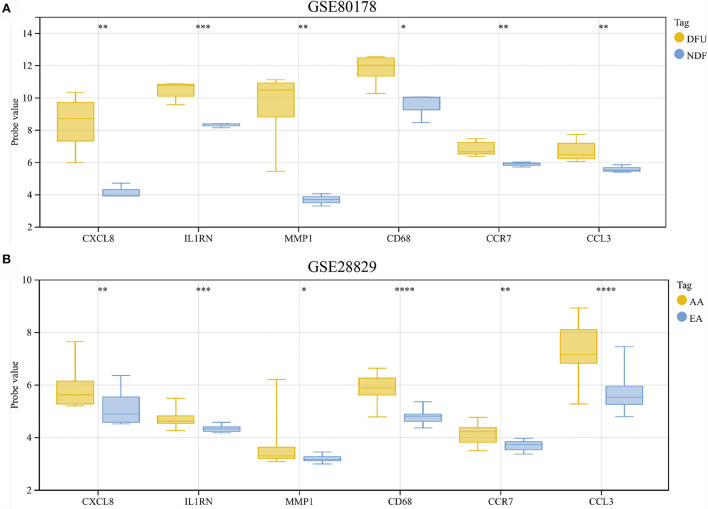
**(A,B)** Hub genes expression in the GSE80178 and GSE28829 datasets. *p < 0.05; **p < 0.01; ***p < 0.001; ****p < 0.0001.

The results showed that compared with NDF, all hub genes were significantly up-regulated in DFU (Figure 8A). Similarly, the expression of these genes in AA was also higher than in EA (Figure 8B).

## Discussion

DFU and PAD possess some common pathogenic factors and accumulated evidence suggest that the developing risk of DFU is associated with PAD, but the detailed molecular mechanisms remain unclear. In this study, bioinformatics method was used to explore the common DEGs in DFU and PAD patients, and the potential signaling pathway and hub genes which involved in the interaction between DFU and PAD were identified. 91 overlapping up-regulated genes and 76 overlapping down-regulated genes were found. KEGG enrichment analysis indicated that up-regulated genes were mainly involved in cytokine-cytokine receptor interaction, viral protein interaction with cytokine and cytokine receptor, IL17 signaling pathway and chemokine signaling pathway. These results strongly indicate that chemokine and cytokine are jointly involved in the occurrence and development of these two diseases. By PPI network constructing, six hub genes (CXCL8, IL1RN, MMP1, CD68, CCR7 and CCL3) were identified. All of them are chemokine or cytokine, which may play an important role in the development of DFU and PAD.

CXCL8 also named as interleukin-8 (IL8), is a proinflammatory chemokine and mainly expressed by the epithelial cells and macrophages. Released CXCL8 could recruit the neutrophil aggregation in tissues/organs which suffered by injury, infection, or inflammation (18, 19). Recently, the transcriptomic analysis of skin in patients with DFU revealed that the expression of CXCL8 was significant increased compared to the control (20). In the wound exudates of patients with DFU, the expression of CXCL8 was also markedly elevated (21). In addition, previous study proven that the expressions of CXCL8 in patients with coronary artery disease were higher than health controls, and oxidized low-density lipoprotein could enhance the expression of CXCL8 in the CAD patients (22). Szomjak et al. found that the circulating chemokine CXCL8 was elevated in patients with cerebro-/cardio-vascular manifestations (23). Furthermore, CXCL8 involved in the regulatory process of NEAT1, a long non-coding RNAs, on atherosclerosis (24). All the above results indicated that CXCL8 may play an important role in the common pathological process of DFU and PAD.

MMP1 is a member of the matrix metalloproteinases (MMP) family, which mainly function as the regulator in the breakdown of extracellular matrix in various physiological processes (25, 26). Luanraksa et al. explored the role of MMP1, MMP9, and TIMP1 in patients with DFU. They found that the concentration of MMP1 was higher in good healers of wound healing degree of patients with DFU, and the MMP1 level possessed the potential predictive effect on the wound healing of DFU patients (27). The above same results were also found by Muller et al. in their experiments (28). Recently, Theocharidis et al. conducted a single-cell RNA sequencing in the skin of patients with DFU, and the results showed that MMP1 was markedly overexpressed in the DFU patients with healing wounds (29). In addition, elevated expression of MMP1 was found in human varicose veins, and MMPs could directly involved in the pathophysiology of many arterial and venous disorders (30). Wigren et al. explored the expression of tissue degradation markers in systemic lupus erythematosus (SLE) patients with or without cardiovascular disease, the results showed that the level of MMP1 in SLE patients with cardiovascular disease was higher than that in SLE patients without cardiovascular disease (31). These evidences indicated that MMP1 may participate in the common pathological process of DFU and PAD.

Furthermore, McDermott et al. explored the association of IL1RN level and the risk of PAD in 2 Italian communities, they found that patients with PAD had the higher level of IL1RN compared to patients without PAD (32). No available studies to identify the role of IL1RN in the development of DFU. In addition, the role of CD68, CCR7, and CCL3 in the pathogenesis of DFU and PAD were also unclear, further data analysis should be conducted to explore these issues.

We must acknowledge the limitations of this study. This is a microarray data analysis study that has not been experimentally validated. It is necessary to conduct further basic and clinical research to explore the changes of these hub genes and signaling pathways, especially those genes that have not been reported to be associated with these two diseases, in order to gain a deeper understanding of the crosstalk between the two diseases.

## Conclusions

In summary, this study investigated the DEGs in DFU and PAD, and the potential common molecular mechanism in the developing risk of DFU and PAD by the bioinformatics method. By PPI network constructing, six hub genes (CXCL8, IL1RN, MMP1, CD68, CCR7 and CCL3) were identified. Based on these results from data analysis, the systemic clinical researches on patients and fundamental research on model animals should be conducted in further studies to clarify the association between DFU and PAD, and the detailed common molecular mechanism.

## Data availability statement

The original contributions presented in the study are included in the article/Supplementary material, further inquiries can be directed to the corresponding authors.

## Author contributions

WS and DY developed a major research plan. JZ, WZ, and XC analyze data, draw charts and write manuscripts. The final manuscript read and approved by all authors. All authors contributed to the article and approved the submitted version.

## Funding

This work was supported by the National Natural Science Foundation of China (32071238), Young Talent Program of China National Nuclear Corporation (CNNC2021136), Natural Science Project of Chengdu Medical College (CYZYB21-07 and CYZZD20-01), Medical Research Project of Chengdu 2021 (2021085) and Natural Science Foundation of Sichuan Province (2020YJ0194).

## Conflict of interest

The authors declare that the research was conducted in the absence of any commercial or financial relationships that could be construed as a potential conflict of interest.

## Publisher's note

All claims expressed in this article are solely those of the authors and do not necessarily represent those of their affiliated organizations, or those of the publisher, the editors and the reviewers. Any product that may be evaluated in this article, or claim that may be made by its manufacturer, is not guaranteed or endorsed by the publisher.

## Supplementary material

Supplementary Table S1 Supplementary The details of the overlapping DEGs.

## Abbreviations

DFU: diabetic foot ulcer
PAD: peripheral artery disease
GEO: Gene Expression Omnibus
GEO: Gene Expression Omnibus
GO: Gene ontology
KEGG: Kyoto Encyclopedia of Genes and Genomes
KOBAS: KEGG Orthology Based Annotation System
DEGs: differentially expressed genes
PPI: protein-protein interaction
TF: transcription factor
NDF: non-diabetic foot skin
AA: arteries with atherosclerotic lesions
CA: control artery samples
FC: fold change
KOBAS: KEGG Orthology Based Annotation System
MCODE: molecular complex detection technology
TRRUST: Transcriptional Regulatory Relationships Unraveled by Sentence-based Text mining
EA: early atherosclerotic plaque samples
SLE: systemic lupus erythematosus.
