# Supplementary material for: Data mining reveal the association between diabetic foot ulcer and peripheral artery disease
Source: Front Public Health. 2022 Aug 18;10:963426. doi: 10.3389/fpubh.2022.963426 (PMC9433977; doi:10.3389/fpubh.2022.963426)
Supplement: Supplementary Table S1 — Supplementary The details of the overlapping DEGs. [file Table_1.DOCX]

| **Gene symbol** | **Expression** | **GSE80178**  **Log2(FC)** | **GSE100927**  **Log2(FC)** |
| --- | --- | --- | --- |
| PSTPIP1 | up | 0.651891481 | 0.943640143 |
| IGF2BP3 | up | 0.691649583 | 0.702965925 |
| CCDC88B | up | 0.785559722 | 0.758931489 |
| RELT | up | 0.890798889 | 0.656388856 |
| HS3ST1 | up | 1.240863056 | 1.04529362 |
| FABP5 | up | 0.822471667 | 1.527458845 |
| PPBP | up | 1.30960375 | 0.797202143 |
| DCSTAMP | up | 0.800564444 | 1.320584441 |
| IGLL1 | up | 0.656088722 | 1.41408775 |
| IL27RA | up | 0.773138194 | 0.630509371 |
| DUSP18 | up | 0.837313194 | 0.604470363 |
| TBX21 | up | 0.611689778 | 0.67527449 |
| KLHL6 | up | 0.707518611 | 1.118231179 |
| CTSB | up | 0.843064167 | 1.546737192 |
| NOXA1 | up | 0.795658194 | 0.615580088 |
| SRMS | up | 0.914784792 | 0.778818245 |
| IRF7 | up | 0.996077639 | 1.122178022 |
| ASCL2 | up | 0.67303912 | 1.193651606 |
| OLR1 | up | 0.650975556 | 0.622619521 |
| IL21R | up | 0.665950694 | 1.003855382 |
| KRT31 | up | 1.015075926 | 0.588793615 |
| TMEM132A | up | 0.830194583 | 0.623403468 |
| MMP1 | up | 5.7934875 | 1.925706921 |
| MMP12 | up | 2.119508125 | 2.328459938 |
| CCR7 | up | 0.953933542 | 1.10080886 |
| CD68 | up | 2.249865463 | 1.592145546 |
| TP53I3 | up | 0.946042361 | 0.739210547 |
| LRFN4 | up | 0.714926019 | 0.613636113 |
| SDS | up | 0.612222083 | 1.10654554 |
| BLNK | up | 1.170654583 | 1.043970453 |
| HMOX1 | up | 1.546298889 | 1.856856583 |
| SIGLEC15 | up | 0.820044769 | 0.907821019 |
| PLD2 | up | 1.371282639 | 0.616522739 |
| SDSL | up | 0.667838333 | 0.800235838 |
| TREM2 | up | 0.936704028 | 1.525772246 |
| GALNT6 | up | 1.447365278 | 1.434055029 |
| SIRPA | up | 1.014816991 | 1.007489855 |
| NECTIN4 | up | 0.687610556 | 1.003520513 |
| CDT1 | up | 0.747984444 | 0.761941243 |
| VENTX | up | 0.909416389 | 0.880856701 |
| PMAIP1 | up | 1.41631912 | 0.822002263 |
| MAPK13 | up | 1.320005833 | 1.058696947 |
| VMO1 | up | 0.701553611 | 1.30181521 |
| SLAMF7 | up | 2.77712375 | 1.789674892 |
| IL1RN | up | 2.142225833 | 1.481451081 |
| ASIC4 | up | 0.745583444 | 0.950765161 |
| PCYOX1L | up | 0.802106111 | 0.597829236 |
| ADAP2 | up | 2.190406389 | 1.74635644 |
| ADAM8 | up | 1.111633565 | 2.015140244 |
| CSTB | up | 1.7986425 | 1.220592832 |
| MBOAT7 | up | 0.733781944 | 0.596091808 |
| TREM1 | up | 2.847943611 | 1.027583223 |
| LIMK1 | up | 0.741372639 | 0.979688717 |
| MATK | up | 0.819026667 | 1.148765237 |
| UNC5B | up | 0.686238981 | 0.818545224 |
| C16ORF54 | up | 0.659397778 | 1.707366373 |
| UAP1L1 | up | 0.613769167 | 0.916362649 |
| CMIP | up | 0.984535 | 0.632438202 |
| ARID3A | up | 0.695325278 | 0.828488929 |
| TTYH3 | up | 1.083711944 | 0.857231564 |
| CXCR2 | up | 0.628886296 | 0.914823267 |
| DSC2 | up | 3.134167778 | 0.806386138 |
| CXCL8 | up | 4.252733333 | 0.63582455 |
| ECM1 | up | 1.090679722 | 0.791180593 |
| HSPA6 | up | 1.018310694 | 0.618251768 |
| GM2A | up | 1.009118889 | 1.067607752 |
| S1PR4 | up | 1.04336 | 1.270432023 |
| ASPHD1 | up | 0.764075556 | 1.197379553 |
| GCHFR | up | 0.623561389 | 0.81085664 |
| S100A8 | up | 5.290126111 | 0.591089007 |
| OASL | up | 1.718009722 | 1.03634169 |
| DHRS9 | up | 1.939178016 | 1.522898577 |
| STXBP2 | up | 0.873066111 | 0.652183249 |
| COTL1 | up | 0.606061458 | 1.155674461 |
| SH2D3A | up | 0.983378333 | 0.636169109 |
| C15ORF48 | up | 3.533752639 | 1.560577649 |
| IGFBP1 | up | 0.722079444 | 0.786337556 |
| PPIF | up | 2.570684259 | 0.777536798 |
| PAQR5 | up | 2.411458056 | 1.177017061 |
| CA2 | up | 1.346605556 | 1.423400523 |
| AGRP | up | 0.842164618 | 0.589616659 |
| ADAP1 | up | 0.871136111 | 0.635524865 |
| ST14 | up | 1.092595139 | 0.728025945 |
| NRIP3 | up | 1.529454583 | 0.593048091 |
| IL7R | up | 0.784506875 | 1.316298884 |
| CXCL5 | up | 1.392174306 | 0.790438693 |
| PKD2L1 | up | 0.784776944 | 0.969126167 |
| F11R | up | 0.6735925 | 0.66546533 |
| HMGA1 | up | 0.879613889 | 0.635201646 |
| CCL3 | up | 1.129443333 | 2.610225122 |
| SPOCD1 | up | 0.801441389 | 0.809612539 |
| FIBIN | down | -1.354436667 | -1.110302835 |
| ANGPTL5 | down | -1.489177685 | -0.749881045 |
| ZEB1 | down | -2.100406389 | -0.830912069 |
| VGLL3 | down | -1.68796691 | -0.780861599 |
| ITGB1BP2 | down | -0.610104861 | -0.767645749 |
| MAOA | down | -0.796735694 | -0.606311514 |
| C3 | down | -2.368078955 | -1.417478434 |
| BCHE | down | -1.479596574 | -0.60972731 |
| HLF | down | -1.837598028 | -0.676752084 |
| HEXIM1 | down | -1.471878333 | -0.657174565 |
| VIT | down | -1.276336111 | -1.048643917 |
| OMD | down | -3.066138981 | -0.703749817 |
| SVIL | down | -0.895974028 | -0.629267474 |
| PDGFD | down | -2.331869722 | -0.62356074 |
| NTN4 | down | -1.278621944 | -0.591534541 |
| PHGDH | down | -1.5749725 | -0.944969313 |
| NPY1R | down | -2.050428194 | -0.835924192 |
| FHL5 | down | -2.016096389 | -1.291961089 |
| TSPAN2 | down | -1.424160583 | -0.675013386 |
| WEE1 | down | -1.286846389 | -0.600646197 |
| ANGPTL1 | down | -4.48236875 | -0.852274271 |
| ASPN | down | -2.128337083 | -0.664770851 |
| OGN | down | -2.127051111 | -0.920510215 |
| AASS | down | -1.357700833 | -0.590611913 |
| APOD | down | -4.497468333 | -1.803532014 |
| PID1 | down | -0.949082778 | -0.634792294 |
| GAS1 | down | -0.957077315 | -0.660874334 |
| GFRA1 | down | -1.109083056 | -0.705681779 |
| CPE | down | -1.380265833 | -0.649621321 |
| GSTM5 | down | -2.09444625 | -0.78326747 |
| DCN | down | -3.086052083 | -0.877991802 |
| CCN5 | down | -1.596555 | -0.675195429 |
| PCOLCE2 | down | -1.014668194 | -0.940503749 |
| ROR1 | down | -1.832805417 | -0.8209659 |
| AOX1 | down | -1.407170417 | -1.488627898 |
| LPP | down | -1.147512917 | -0.926478691 |
| FGL2 | down | -2.492035556 | -0.784356421 |
| CXCL14 | down | -1.43990162 | -2.911771231 |
| PKD2 | down | -1.728724028 | -0.68837811 |
| PLN | down | -1.593530139 | -1.418509625 |
| FOXC1 | down | -0.853160116 | -0.81724442 |
| TSPAN8 | down | -0.951174167 | -1.065403858 |
| MYEF2 | down | -1.121702778 | -1.000521727 |
| ANGPTL7 | down | -2.13947125 | -1.131557629 |
| FBLN1 | down | -0.866344931 | -1.489501913 |
| TMEM47 | down | -2.778640806 | -1.118135856 |
| ACKR4 | down | -1.206505972 | -1.033466878 |
| ELN | down | -0.793290278 | -0.999503583 |
| TPM1 | down | -1.475230972 | -0.796927344 |
| RNF115 | down | -1.116566111 | -0.7165906 |
| TNS1 | down | -1.934408611 | -0.712779588 |
| GRB14 | down | -1.102466944 | -0.770067613 |
| C7 | down | -2.998685139 | -0.887341615 |
| CPED1 | down | -1.878075556 | -0.599893847 |
| FERMT2 | down | -0.964746944 | -0.906316089 |
| MAOB | down | -1.715056375 | -0.700096764 |
| BTC | down | -2.373176944 | -0.995120471 |
| LRCH2 | down | -0.999369167 | -0.73287232 |
| OSR1 | down | -0.616459444 | -0.708059909 |
| AVPR1A | down | -0.738635417 | -0.938237522 |
| NEXN | down | -1.359933472 | -1.04895437 |
| FRK | down | -1.534723194 | -0.686228679 |
| ITM2A | down | -1.696248083 | -0.868765117 |
| MAP1B | down | -2.404688611 | -0.831557489 |
| HEY2 | down | -1.012081667 | -0.716079896 |
| CSRP2 | down | -1.39426662 | -0.877141366 |
| PDLIM3 | down | -0.973581667 | -0.774035177 |
| CCN3 | down | -1.672521806 | -0.93231641 |
| HSD11B1 | down | -1.160395972 | -0.833408678 |
| CASQ2 | down | -1.67102 | -1.278955819 |
| LPAR1 | down | -0.617670278 | -0.799004696 |
| ADAMTSL3 | down | -1.390619583 | -0.690962306 |
| SFRP1 | down | -1.073675139 | -1.345539992 |
| PARM1 | down | -2.105109688 | -1.052359686 |
| SPART | down | -0.889787639 | -0.659894437 |
| FBLN5 | down | -2.402907917 | -1.08376696 |
